# Parallel Evolutionary Dynamics of Adaptive Diversification in *Escherichia coli*

**DOI:** 10.1371/journal.pbio.1001490

**Published:** 2013-02-19

**Authors:** Matthew D. Herron, Michael Doebeli

**Affiliations:** 1 Department of Zoology, University of British Columbia, Vancouver, British Columbia, Canada; 2 Department of Mathematics, University of British Columbia, Vancouver, British Columbia, Canada; University of Colorado, United States of America

## Abstract

The causes and mechanisms of evolutionary diversification are central issues in biology. Geographic isolation is the traditional explanation for diversification, but recent theoretical and empirical studies have shown that frequency-dependent selection can drive diversification without isolation and that adaptive diversification occurring in sympatry may be an important source of biological diversity. However, there are no empirical examples in which sympatric lineage splits have been understood at the genetic level, and it is unknown how predictable this process is—that is, whether similar ecological settings lead to parallel evolutionary dynamics of diversification. We documented the genetic basis and the evolutionary dynamics of adaptive diversification in three replicate evolution experiments, in which competition for two carbon sources caused initially isogenic populations of the bacterium *Escherichia coli* to diversify into two coexisting ecotypes representing different physiological adaptations in the central carbohydrate metabolism. Whole-genome sequencing of clones of each ecotype from different populations revealed many parallel and some unique genetic changes underlying the derived phenotypes, including changes to the same genes and sometimes to the same nucleotide. Timelines of allele frequencies extracted from the frozen “fossil” record of the three evolving populations suggest parallel evolutionary dynamics driven at least in part by a co-evolutionary process in which mutations causing one type of physiology changed the ecological environment, allowing the invasion of mutations causing an alternate physiology. This process closely corresponds to the evolutionary dynamics seen in mathematical models of adaptive diversification due to frequency-dependent ecological interactions. The parallel genetic changes underlying similar phenotypes in independently evolved lineages provide empirical evidence of adaptive diversification as a predictable evolutionary process.

## Abstract 2

The divergence of *Escherichia coli* bacteria into metabolically distinct ecotypes has a similar genetic basis and similar evolutionary dynamics across independently evolved populations.

## Author Summary

The causes and mechanisms of evolutionary diversification are central issues in biology. There is well-established theory that predicts that adaptive diversification can arise because of ecological interactions between individuals, such as competition or predation, but there are no empirical examples in which this process has been observed at the genetic level. We documented the genetic basis of adaptive diversification resulting from competition for resources in populations of the bacterium *Escherichia coli*. The populations diversified into two coexisting ecotypes representing different physiological adaptations. We found that similar but independently evolved phenotypes often shared mutations in the same gene and, in four cases, shared identical mutations at the same nucleotide position. Timelines of allele frequencies extracted from the frozen “fossil record” of three evolving populations showed parallel evolutionary dynamics, suggesting that mutations causing one type of physiology changed the ecological environment and allowed invasion of mutations causing an alternate physiology. The results provide empirical evidence of adaptive diversification as a predictable evolutionary process.

## Introduction

The causes and mechanisms of diversification are central issues in evolutionary biology. Explanations that involve the splitting of an ancestral population into geographically or otherwise isolated populations (allopatric diversification) have historically been favored because of theoretical difficulties with sympatric diversification (i.e., diversification without isolation) [1]–[5]. In the last 15 years, though, two major developments have increased the attractiveness of sympatric explanations. First, models of sympatric diversification have largely overcome earlier theoretical objections, showing that sympatric diversification can occur due to frequency-dependent selection under a wide range of conditions [6]–[9]. Second, empirical evidence from both laboratory experiments [10]–[19] and field studies [20]–[23] suggests that diversification can occur in sympatry, sometimes on time scales of hundreds of generations, and that such diversification may be an important source of biological diversity.

Sympatric diversification can be driven by frequency-dependent selection in a process called adaptive diversification and under conditions that may be quite general [7]–[9],[24]. This process can be described by the theoretical framework of adaptive dynamics [6],[25],[26]. A crucial component of this framework is the concept that the environment a population experiences, and that drives its evolutionary dynamics, depends in part on the phenotypic distribution of the population itself and the resulting ecological dynamics. Adaptive diversification occurs through evolutionary branching [6], a process in which selection drives a population to a point in phenotype space at which selection becomes disruptive. At this point, the population diverges into two lineages, which may continue to diverge.

In general, the problem of adaptive diversification and speciation is 2-fold: on the one hand, one wants to identify the ecological conditions that lead to disruptive selection and evolutionary branching, and on the other hand, one wants to understand the mechanisms interrupting gene flow between ecologically diverging subpopulations. Both of these aspects of adaptive diversification have been studied extensively in the theoretical literature (e.g., [7]–[9]). Here we experimentally address the first of these issues using asexual organisms, in which mating does not lead to recombination between diverging subpopulations, and which are therefore ideally suited to study the ecological conditions generating the frequency dependence necessary for adaptive diversification. Indeed, adaptive diversification has been documented in microbial evolution experiments [11],[12],[27]–[31] in which well-mixed populations of *Escherichia coli* bacteria founded with a single genotype repeatedly evolve two metabolically distinct phenotypes. When grown in well-mixed serial batch cultures in medium with glucose and acetate as carbon sources, *E. coli* cells preferentially metabolize glucose and excrete acetate until the glucose is depleted and then undergo a diauxic switch to acetate consumption [32]. In several populations evolving in these conditions for more than 1,000 generations, two coexisting phenotypes emerged that differ in their diauxic lag—that is, in the time required to switch to acetate metabolism: the slow switcher (SS) has a longer diauxic lag than that of the fast switcher (FS) [11],[28]. These two phenotypes reflect a tradeoff in carbohydrate metabolism: SS strains grow more quickly than FS strains when glucose is abundant, but are unable to efficiently catabolize acetate, while FS strains continue to grow rapidly on acetate after glucose is depleted [28]. The evolution of the FS and SS phenotypes in multiple replicate lines is a striking example of convergence at the phenotypic level, suggesting a deterministic adaptive process.

However, the evolutionary branching predicted by adaptive dynamics models necessarily involves changing selective pressures. Therefore, the similar outcomes of diversification across replicate populations are qualitatively different from parallel adaptation to a fixed adaptive landscape. Rather, in this case, the entire process of genetic change leading to environmental change and new selective pressures that in turn cause further genetic change has occurred in parallel. This suggests that not only the outcome of evolution is parallel but the evolutionary dynamics as well.

In spite of phenotypic evidence for adaptive diversification, there is limited information available on the genetic changes underlying this process. In fact, to our knowledge there are no examples of sympatric diversification for which the underlying genetics have been fully described. In the FS and SS example, the degree to which the similar, independently evolved phenotypes reflect similar underlying genetics in different populations is unknown. This has implications for the genotype–phenotype map: Are there few genetic ways to produce FS and SS phenotypes or many? Also unknown is the degree to which the similar evolutionary outcomes reflect similar evolutionary dynamics; the results of previous studies suggest that the degree of similarity in the type, order, and timing of adaptive changes across independently evolving populations varies widely (e.g., [33]–[35]). This in turn has implications for the degree of determinism in the evolutionary dynamics: Are there many paths or few that lead to similar phenotypic (and possibly genetic) outcomes? And are the changing selective pressures predicted by adaptive dynamics models reflected in genetic changes leading to new selective pressures that in turn cause further genetic change? If such a pattern is present in multiple replicate lines, this would provide evidence that not only the outcome of evolution is predictable, but the evolutionary dynamics as well.

To trace the dynamics of genetic change underlying adaptive diversification, we combined sequencing of FS and SS clones isolated near the end of the evolution experiment with sequencing of whole-population samples from time points in the frozen (“fossil”) record of the experiment. We sequenced two FS clones, two SS clones, and 16 time point samples for each of three replicate evolution experiments (called populations 18, 19, and 20 [28]). Sequencing of SS and FS clones allowed us to identify mutations associated with the phenotypes of interest, and sequencing of whole-population samples from the fossil record of the experiments allowed us to trace the origin, increase, and (occasionally) extinction of these and other mutations. Finally, comparing these results across three independently evolved populations allowed us to assess the degree to which a similar ecological setting led to similar evolutionary dynamics and outcomes (i.e., the degree of determinism).

## Results

**Figure 1:**
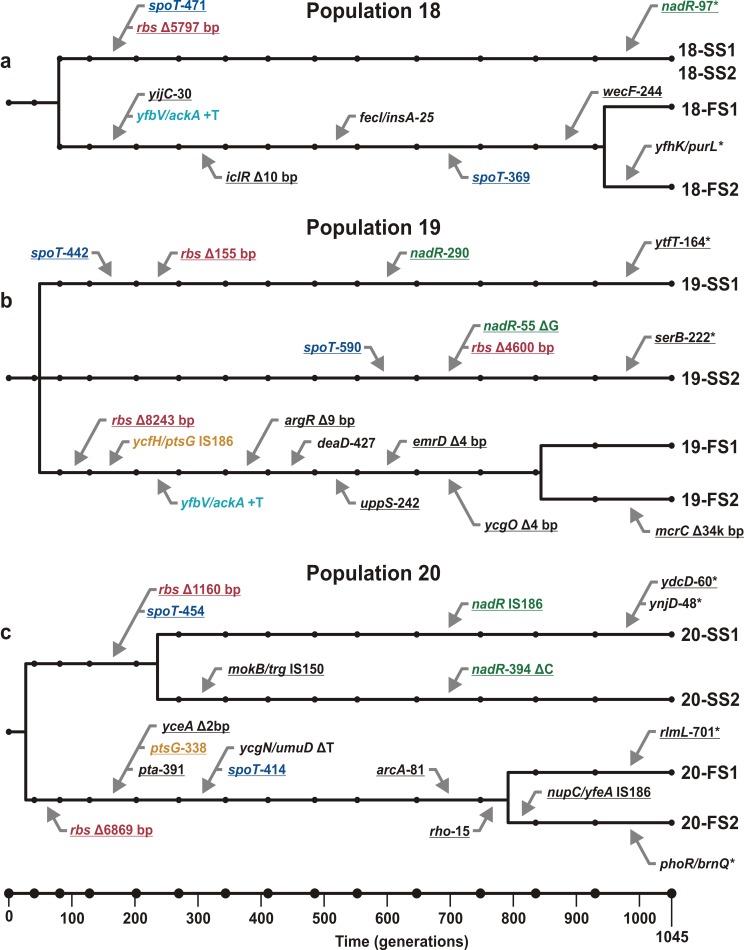
Mutations found in sequenced clones from generation 1045 and inferred genealogical relationships among the clones. (a) population 18. (b) population 19. (c) population 20. Black circles indicate the time point samples from the frozen “fossil” record. Δ, deletion; +, insertion. Numbers following gene names indicate the affected codon within the gene. Two gene names separated by a forward slash (e.g., *yfbV*/*ackA*) indicate that the mutation is in the intergenic region between the indicated genes. Underlined gene names indicate nonsynonymous changes (including indels in coding regions). Colored gene names (other than black) indicate changes in or upstream of the same gene. Timing of mutations and divergences were inferred from the fossil record: a mutation found in a clone was assumed to have arisen at the midpoint between the first time step at which the mutation was detected in the fossil record and the previous time step; divergences between clones are assumed midway between the last mutation they share and the first mutation they do not share. Because of limitations on time resolution and minimum detectable frequency, timing of all such events should be viewed as approximations. Mutations found in clones but not in time point samples are assumed to have occurred near the end of the experiment and are marked with asterisks (*).

Sequencing the SS and FS clones revealed striking similarities in the genetic changes underlying the derived phenotypes across the three replicate populations (Figure 1). Each of the SS clones carried a mutation in *spoT*, a deletion of part or all of the ribose operon (*rbs*), and a mutation in *nadR* (Figure 1). One or two additional mutations appeared in some SS clones, but these were not shared between clones. No mutations were fixed in any of the three replicate populations, and in no case was any specific genetic change shared between FS and SS clones. In population 19, the two SS clones did not share any mutations (Figure 1b), indicating that they evolved independently from the ancestral strain (although each clone has a mutation in *spoT*, *nadR*, and *rbs*). Thus, the six sequenced SS clones represent four separate origins of the SS phenotype, all of which evolved parallel changes to the same three loci.

Each of the FS clones carried 6–10 mutations relative to the ancestral strain, most of which were shared between the two clones from each population (Figure 1). Assuming a single origin for each mutation, we infer that these shared mutations occurred before the two sequenced clones last shared a common ancestor. Phenotypically, the FS type represents a novel metabolic strategy, while the SS type is more similar to the ancestral strain [11],[27],[28],[30], and this difference is reflected in the underlying genetics. In all three populations, the FS clones are more genetically distant from the ancestor than the SS clones (paired *t* test, *n* = 4 independent comparisons, two-tailed *p* = 0.0008). FS clones from different populations are also more genetically dissimilar than SS clones from different populations: in contrast to *spoT*, *rbs*, and *nadR* in the SS clones, there were no genes that carried mutations in the FS clones from all three populations.

**Figure 2:**
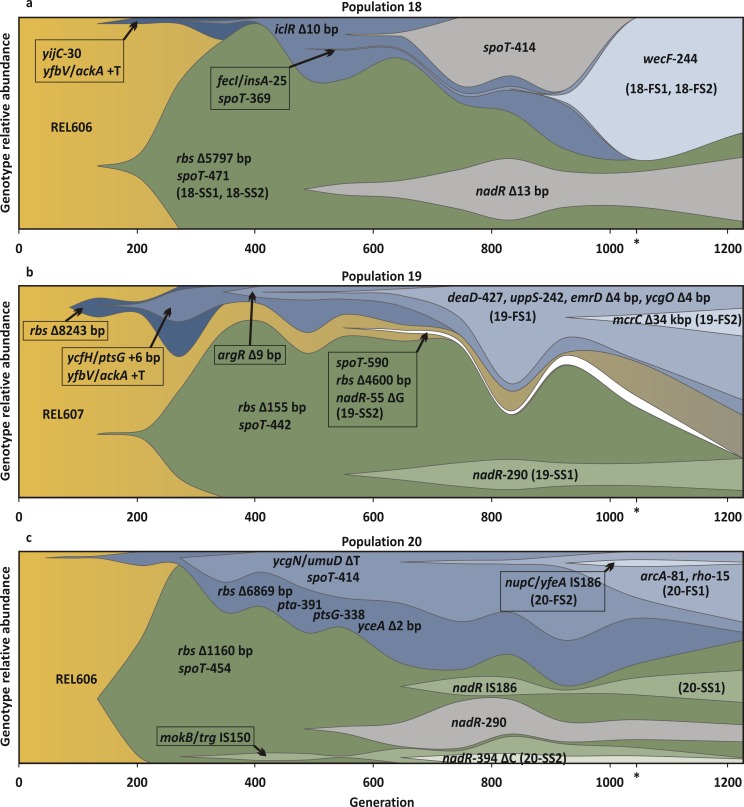
Dynamics of the frequencies of mutations detected in the fossil record of three evolving populations. Shades of blue (above) indicate the mutations associated with FS clones as identified in Figure 1, and shades of green (below) indicate the mutations associated with SS clones as identified in Figure 1 (mutations with a * in Figure 1 are not shown, because their frequency was not high enough to be detected in the time point samples). Gold indicates ancestral strains (which may include mutations not associated with any sequenced clone). The white region in (b) indicates an independent origin of the SS phenotype. Mutations within a lineage are cumulative—that is, mutations corresponding to lighter regions appear in the genetic background corresponding to the darker regions in which the lighter regions are nested. For example, in population 20 the first mutations to appear in the SS lineage were an 1,160 bp deletion in the *rbs* operon and a substitution in codon 454 of *spoT*. An IS*150* insertion in the intergenic region between *mokB* and *trg* appeared on this background around generation 300 and remained at low frequency for the rest of the experiment. Around generation 650, a single bp deletion in codon 394 of *nadR* appeared on the *rbs* Δ1160 bp+*spoT*-454+*mokB*/*trg* IS*150* background. Grouping of mutations into lineages was based on their presence together in sequenced clones (in this case 20-SS2), and their order of appearance was inferred from the time point sample in which each was first detected. In addition, three mutations not found in any of the sequenced clones but whose association with SS and FS can be inferred (explained in SI) are shown in gray [FS-associated *spoT*-414 in (a), and SS-associated *nadR*-235 in (a) and *nadR*-290 in (c)]. For visual clarity, mutations of similar frequency within a lineage have been lumped together and their frequencies averaged. See Figures 3–5, S1, and S2 for the frequencies of individual mutations. The * on the time axis indicates the time when the sequenced clones (Figure 1) were extracted.

**Figure 3:**
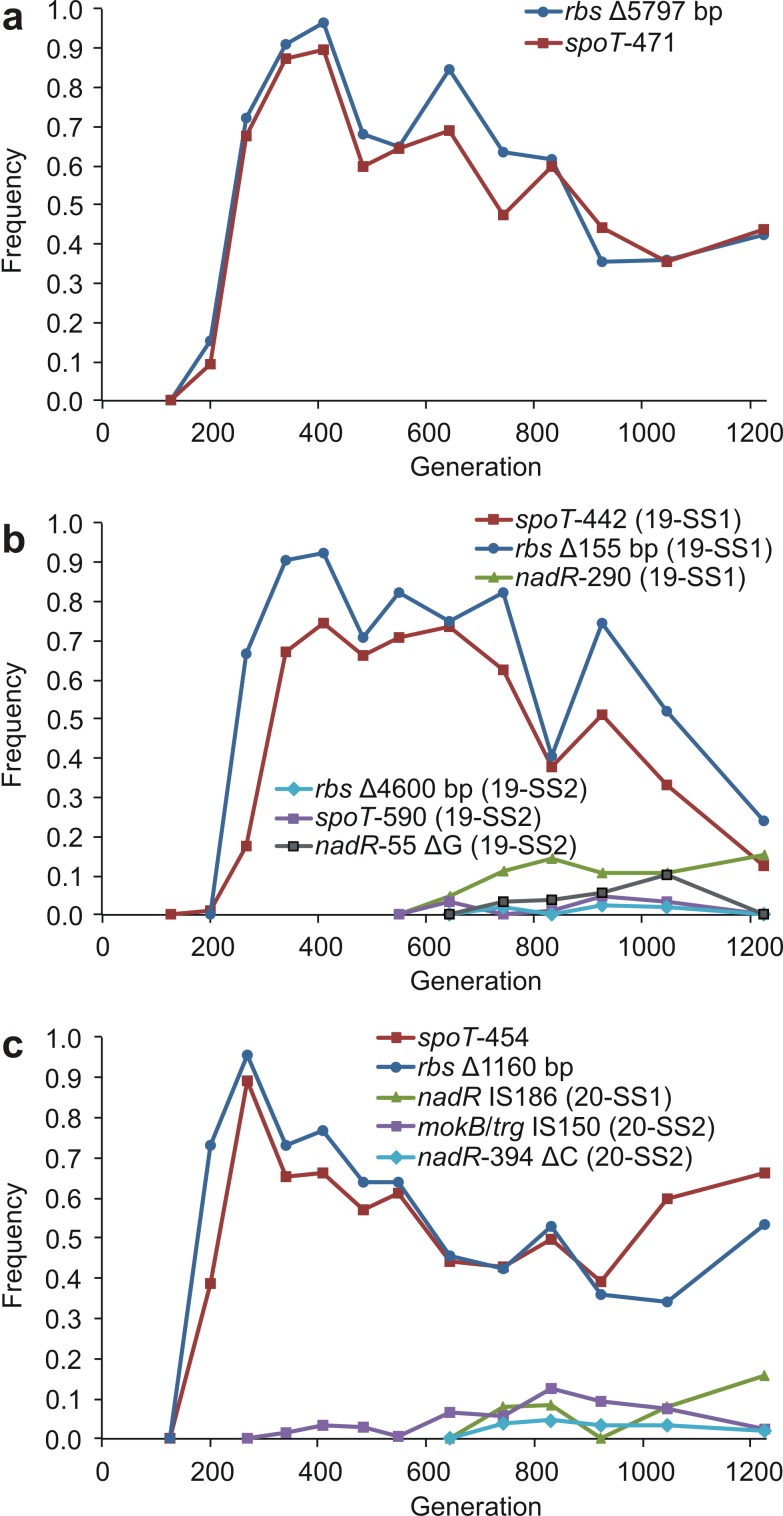
Dynamics of frequencies of mutations found in the SS clones. (a) population 18. (b) population 19. (c) population 20. Δ, deletion; +, insertion. Numbers following gene names indicate the affected codon within the gene. Two gene names separated by a forward slash (e.g., *mokB/trg*) indicate that the mutation is in the intergenic region between the indicated genes. Mutations shown are in both SS clones from the population except where indicated otherwise.

**Figure 4:**
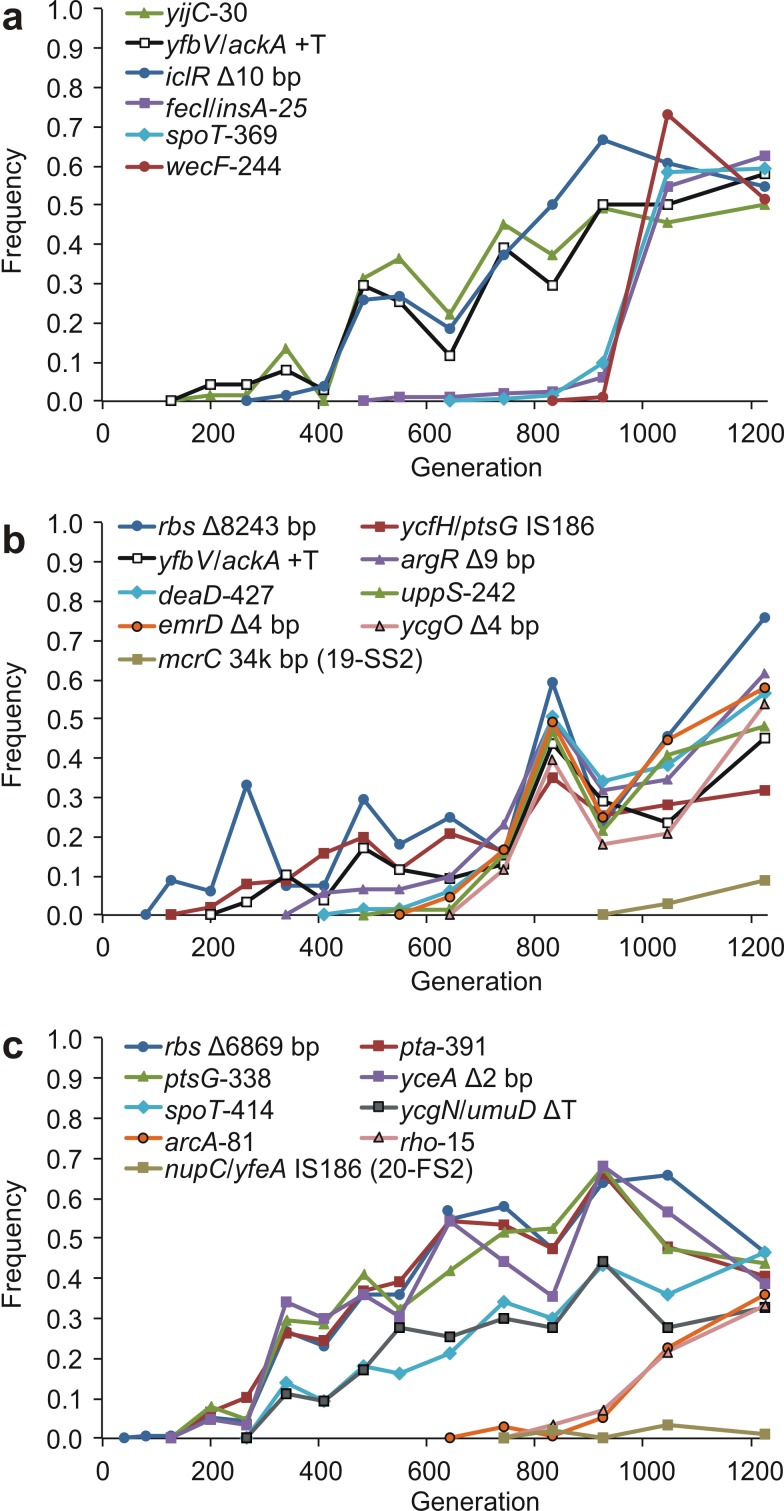
Dynamics of frequencies of mutations found in the FS clones. (a) population 18. (b) population 19. (c) population 20. Δ, deletion; +, insertion. Numbers following gene names indicate the affected codon within the gene. Two gene names separated by a forward slash (e.g., *yfbV/ackA*) indicate that the mutation is in the intergenic region between the indicated genes. Mutations shown are in both FS clones from the population except where indicated otherwise. The +T insertion between *yfbV* and *ackA* is identical in populations 18 and 19.

Timelines of allelic invasions in the SS and FS lineages are shown in Figures 2–4. Figure 2 summarizes the evolutionary dynamics unfolding in each of the three evolution experiments, and Figures 3 and 4 show the frequencies of the mutations found in the various SS and FS endpoint clones over time. These timelines suggest that each ecotype affected the other's evolution by altering the available ecological opportunities. In all three evolving populations, nonsynonymous SS-associated *spoT* and *rbs* mutations were the first to reach high frequency and likely increased the degree of specialization on glucose [36],[37]. In population 18, for which the timeline of metabolic phenotypes has been documented [28], the rapid rise of these mutations corresponds very well with the increase in the mean switching lag shown in Figure 1B of Spencer et al. [28]. Similarly, in population 20, SS bacteria were present by generation 200 [31]. In both cases, *spoT* and *rbs* were the only SS-associated mutations present when the SS phenotype was first detected, so one or both of these mutations must have caused the SS phenotype. It is known that *spoT* mutations can confer a substantial advantage by reducing the lag phase before exponential growth on glucose and by increasing the maximum growth rate on glucose, both of which presumably occur through partial deactivation of the stringent stress response [37],[38]. This may in turn make it harder for the cells to switch to acetate consumption after glucose is exhausted, and hence cause the SS phenotype.

Due to an IS*150* element immediately upstream of the *rbs* operon, deletions of all or part of *rbs* occur at high frequency (∼5×10^−5^ per cell generation) in the ancestral *E. coli* strains used in our evolution experiments and provide a ∼1%–2% fitness advantage in glucose minimal medium [36]. Since *rbs* deletions were also the first mutations to occur in two of the three FS lineages (Figure 1b, c), it is likely that *rbs* deletions alone do not cause either the SS or the FS phenotype, but rather that *rbs* deletion mutants were a common genetic background early in the experiment and that the mutations causing the SS and most FS phenotypes occurred on this background.

By generation 342, the frequency of SS-associated *spoT* and *rbs* mutations was high (>65%) in all three populations (Figure 3). If either or both of these mutations are responsible for an increase in acetate lag (as must be the case in population 18), their increased frequency would have caused a change in the daily regime of nutrient concentrations in the experimental environment, namely that more acetate was available later in the growth phase. The first FS-associated mutations began to rise in frequency at this time (Figures 2 and 4). This wave of invasion involved a different set of genes in each population, but some evidence of parallelism is apparent here as well: the mutations increasing at this time included an identical insertion in the *yfbV*/*ackA* intergenic region in populations 18 and 19, and different mutations affecting the *ptsG* gene in populations 19 and 20.

In all three populations, the first FS-associated mutations to reach appreciable frequency included ones in or upstream of genes related to acetate utilization and excretion and glucose metabolism. These mutations appeared either in the remaining ancestral genetic background or in *rbs* deletion mutants and led to coexistence between the SS and FS lineages that persisted until the end of the evolution experiments. These early FS-associated mutations occurred upstream of *ackA* in populations 18 and 19, in *iclR* in population 18, in *pta* in population 20, and in or upstream of *ptsG* in populations 19 and 20 (Figure 2). The timing of these invasions, which in all three populations only reached appreciable frequencies after SS-associated mutations had reached high frequency, is consistent with FS-like phenotypes evolving as an adaptation to the novel ecological niche of greater acetate availability generated by increased glucose specialization of the SS. These early FS invasions thus generated the basic SS-FS-polymorphism that persisted to the end of the evolution experiment. Experimental evidence demonstrates that the long-term coexistence of FS and SS is due to frequency-dependent interactions [28]–[31]. Again, in population 18 the correspondence with phenotypic change is conspicuous: clones with a short acetate lag were first detected around the same time (ca. generation 500, Figure 1B in Spencer et al. [28]) at which the first three FS-associated mutations reached appreciable frequency: a nonsynonymous substitution in *yijC*, an insertion upstream of *ackA* (*yfbV*/*ackA*+T), and a 10 bp deletion in *iclR* (Figures 2 and 4). Thus one or more of these must have produced the FS phenotype. By the same logic, one or more of the four FS-associated mutations present in population 20 when FS were first detected at generation 200 (*rbs*, *pta*, *ptsG*, and *yceA*) must be sufficient to produce the FS phenotype.

The functions of some genes in the initial FS invasions suggest their involvement in similar phenotypic changes across populations. The *yfbV/ackA* insertion in populations 18 and 19 affects a potential transcriptional recognition sequence of the global fermentation activator *arcA* upstream of *ackA* [39], suggesting that this mutation affects *ackA* expression, and hence acetate metabolism. In population 20, a mutation in *pta* rose in frequency at about the same time (Figure 4c), and all six sequenced FS clones bear one of these two mutations. Since *ackA* and *pta* catalyze subsequent reactions in the pathway of acetate utilization and excretion (acetate↔acetylphosphate and acetylphosphate↔acetyl-CoA, respectively), these two mutations may have similar metabolic effects.

The function of *ackA* as an important regulator of acetate metabolism and the independent origin of the identical *yfbV*/*ackA* mutation in populations 18 and 19 strongly suggest that this intergenic substitution is at least partially responsible for the reduced acetate lag in the FS clones (although FS in population 20 has a different genetic basis). Similarly, *iclR* is a regulator of the acetate operon *aceBAK*, and in an experimental population not included in this study, an insertion in *iclR* acting as a stop codon was previously shown to be partly responsible for the FS phenotype by derepressing the acetate operon [27]. This suggests that the *iclR* deletion in population 18 has contributed to the FS phenotype as well. Finally, *yijC*, a repressor of genes involved in fatty acid biosynthesis [38], could play a role in the FS phenotype by altering the relative amounts of acetyl-CoA used in fatty acid biosynthesis and in the citric acid cycle.

In population 20, a mutation in *ptsG* was one of the first FS-associated mutations to invade (Figure 4c), while in population 19, an IS*186* insertion sequence appeared in the intergenic region upstream of *ptsG* around the same time, potentially disrupting its transcriptional regulation. The enzyme encoded by *ptsG*, a glucose-specific PTS permease, is involved in the uptake of glucose and its transport across the cell membrane [40], and disruption or down-regulation of these functions would be consistent with the FS phenotype.

**Figure 5:**
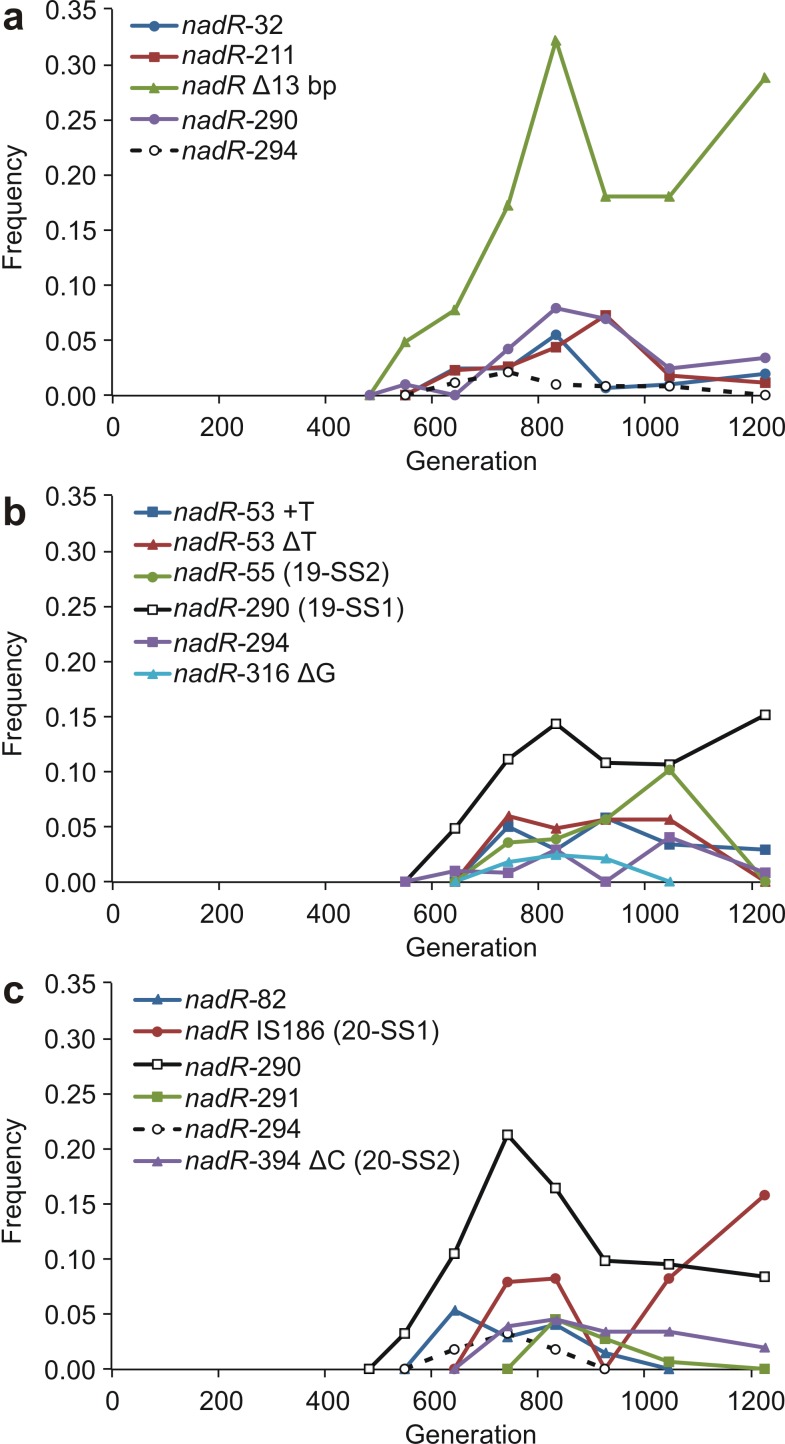
Frequencies of mutations in the nadR gene. (a) population 18. (b) population 19. (c) population 20. Δ, deletion; +, insertion. Numbers following gene names indicate the affected codon within the gene. FS, SS1, etc. in parentheses indicate which clones (if any) have the mutation. The *nadR*-290 mutation is identical in populations 19 and 20; *nadR*-290 in population 18 is a different mutation in the same codon. The *nadR*-294 mutation is identical in populations 18 and 20; *nadR*-294 in population 19 is a different mutation in the same codon. Note that the highest frequency on the *y*-axis is 0.35.

After FS mutations had risen to intermediate frequencies (>0.15), several SS-associated *nadR* mutations appeared at detectable frequencies in each of the three populations (Figures 1, 2, and 5; Text S1). The proliferation of these mutations (≥5 in each population) after generation 500 is striking since no *nadR* mutations were present at detectable frequency before this time. *nadR* plays an important role in many metabolic pathways, including growth on carbohydrates [41],[42], and the observed mutations show a surprising degree of parallelism. The highest-mean frequency *nadR* mutation in population 20 (*nadR*-290) was identical to that in 19-SS1 in population 19, and a different mutation in the same codon was present in population 18. All three populations also included a mutation in codon 294 of *nadR*, and this was identical between populations 18 and 20 (Figure 5a, c). Thus, a different pair of mutations in these two codons is found in each of the three populations, though each mutation is shared by two populations.

The *nadR* mutation found in both SS clones from population 18 was only detected in a single Illumina read in the time point samples (at generation 482), indicating that it was present at very low frequency. The presence of such a low-frequency mutation in both sequenced clones suggests that it had a phenotypic effect (since we preferentially selected clones that were clearly of the SS phenotype; see Materials and Methods). The protein encoded by *nadR* has both enzymatic and regulatory roles in the NAD biosynthetic pathway and plays important roles in glycolysis and the citric acid cycle. The presence of *nadR* mutations in all six sequenced SS clones and none of the six sequenced FS clones strongly suggests that these mutations are adaptive for the SS, but not the FS, phenotype. It is interesting to note that mutations in *nadR* were found in 12 of 12 experimental *E. coli* populations after 20,000 generations of evolution in glucose minimal medium [42], and that one of these was identical to the *nadR*-290 mutation in populations 19 and 20.

In populations 18 and 20, invasion by SS-associated *nadR* mutants was followed by rapid increases in frequency of a second set of FS-associated mutations (Figure 2). In population 18, a *spoT* mutation identical to that in FS from population 20 (*spoT*-414) increased in frequency only to be replaced by another *spoT* mutation (*spoT*-369) that had previously been present at very low frequency. In population 20, the second set of FS-associated mutations included one in a global regulator (*arcA*) known to increase acetate consumption [31]. It is likely that the FS-associated *arcA* mutation in population 20 affects the expression of *ackA*; if so, one of the phenotypic effects of this mutation may be similar to that of the *yfbV*/*ackA* insertion in populations 18 and 19. This would explain why this mutation has a larger impact on SS clones than on FS clones [31]: if the primary phenotypic effect of the *arcA* mutation is to alter the rates of acetate utilization and/or excretion, the FS-associated *pta* mutation may have made this effect at least partially redundant in population 20.

In addition to the *spoT* mutations associated with FS and SS clones, one other mutation in *spoT* was present at ≥20% frequency at some time in each of the three populations (Figure S1). In populations 18 and 20, this mutation was lost by the end of the experiment. In population 19 this *spoT* mutation increased in frequency near the end of the experiment as the *spoT* mutation associated with 19-SS1 underwent a corresponding decline. The transient *spoT* mutation in population 18 was identical to that associated with the FS clones in population 20 (Figure S1a, c), and hence is likely to be FS-associated. This indicates that mutations in the stringent response can be adaptive for either the SS or the FS phenotype [43]. The phenotype associated with the *spoT*-316 mutation in population 20 is not known.

Several other mutations not associated with any of the FS and SS clones were present at detectable frequencies in each of the three fossil records (Figure S2). A complete list of detected mutations and the samples in which they were found is shown in Table S1.

## Discussion

Microbial evolution experiments are a powerful approach to understanding evolutionary dynamics, combining controlled conditions with the capability for experimental replication to allow strong inferences of causation. In addition, rapid reproduction allows laboratory experiments lasting hundreds or thousands of generations, and cryopreservation allows direct comparisons between ancestors and descendants. The recent rapid advance of nucleic acid sequencing technologies has made whole-genome sequencing feasible for both single microbial strains and whole populations containing a variety of strains. The combination of microbial evolution experiments and next-generation sequencing technologies provides an unprecedented opportunity to observe the temporal dynamics of evolutionary change across the entire genome [44],[45]. Replicating this approach in multiple independent populations can tell us whether adaptive sympatric diversification in independent populations involves similar genetic mechanisms and similar evolutionary dynamics.

Our results revealed both shared and unique genetic mechanisms underlying the evolution of pairs of metabolically distinct ecotypes in different populations. In some cases, similar phenotypes had mutations in different genes (e.g., the *wecF*, *uppS*, and *arcA* mutations in the FS clones from populations 18, 19, and 20, respectively; no mutations in these genes were detected in either clones or time point samples in the other populations). In some cases, mutations affected different codons of the same gene, as in the distinct *spoT* and *nadR* mutations found in the SS clones from all three populations. We also observed different changes to the same codon (e.g., codons 290 and 294 of the *nadR* gene; Figures 2 and 5). Finally, we found four examples of identical genetic changes in different populations: *spoT*-414 (populations 18 and 20; Figure 2), *yfbV*/*ackA* (18 and 19; Figure 2), and two *nadR* codons (290 identical in populations 19 and 20, Figure 2; 294 identical in 18 and 20, Figure 5). Of the 45 mutations shown in Figure 1, 21 (47%) occurred in a nucleotide, codon, or gene that also had a mutation associated with the same ecotype in another population.

The pattern of genetic invasions evident in the fossil records also revealed strikingly similar evolutionary dynamics: in all three evolving populations, SS-associated *spoT* and *rbs* mutations were the first to invade, followed by FS-associated mutations affecting acetate and glucose metabolism, followed by SS-associated mutations in *nadR*, and finally additional FS-associated mutations. In spite of several mutations showing evidence of strong positive selection, such as the SS-associated *spoT* mutations in all three populations, no mutation was fixed in any of the three populations. Many mutations that increased rapidly after their initial appearance later declined in frequency yet were then maintained in the populations at intermediate frequencies.

Apart from genetic drift, two separate (but not mutually exclusive) processes could explain the repeated and parallel invasions and long-term coexistence observed in these three populations. We do not consider genetic drift as an explanation because the large effective population sizes make drift implausible for allele frequency changes greater than a fraction of 1% from one time point sample to the next (see Materials and Methods).

Clonal interference, which involves the coexistence of two or more beneficial mutations on different genetic backgrounds, is one potential explanation. This process allows long transient polymorphisms to be maintained in asexual populations because several different and almost equally beneficial mutations can be present in different subpopulations [46]–[48]. Thus, clonal interference is expected to lead to longer fixation times, elevated levels of polymorphism, and generally more complex evolutionary dynamics in asexual populations such as the ones studied here. One probable example of clonal interference is the replacement of *spoT*-414 by *fecI/insA-25*, *spoT*-369, and *wecF*-244 within the FS lineage in population 18. Around generation 700, the *spot*-414 mutation appeared on the FS background and began to rapidly invade, while the *fecI/insA*-25 and *spoT*-369 mutations remained at low frequency. Before the *spoT*-414 mutation could reach fixation within the FS lineage, though, the *wecF*-244 mutation appeared and quickly replaced all other FS lineages, including that with *spoT-*414.

Another possible explanation for long-term coexistence is the coevolution of diverging phenotypes through environmental feedbacks and frequency-dependent selection. In this scenario, the adaptive landscape changes as metabolic changes in one subpopulation create a new niche, which another subpopulation evolves to fill. Since the only source of environmental change over the course of the experiments was the bacteria themselves, any such changes in the selective regime must have been generated by changes in the genetic, and hence metabolic, makeup of the bacterial populations. Such environmental feedback generates frequency dependence and is at the core of the theory of adaptive diversification [6]–[9]. An example of environmentally mediated negative frequency dependence is the interaction between the SS lineage and the *wecF*-244 containing FS lineage in population 18: the *wecF*-244 mutation invaded the FS lineage rapidly, indicating a strong selective advantage. In the absence of any frequency-dependent interactions, such an advantageous mutation would continue to invade, going quickly to fixation unless another even more advantageous mutation appeared (as in the clonal interference scenario). In this case, though, neither of these explanations is viable: after quickly fixing within the FS lineage, the *wecF*-244 mutation leveled off (or even declined in frequency) in the absence of any new mutations.

Taken by themselves, most of our results could be explained by either clonal interference or reciprocal niche construction. Since both processes can explain the long-term coexistence of multiple lineages, it can be difficult to distinguish between them. However, the populations in this study have also been the subject of numerous previous studies, and this prior work aids substantially in interpreting the current results. When this additional information is taken into account, it is clear that although clonal interference may explain some of the observed dynamics, it is unlikely to explain all of them.

The main reason for this is that we already know from previous experimental analyses that the coexistence between the SS and FS ecotypes involves frequency dependence, at least in populations 18 and 20 (e.g., [28]–[31]). In particular, the polymorphisms between SS and FS lineages that we observed arising early on in the evolution experiments are maintained by selective forces favoring rare ecotypes. For population 20, [31] has explicitly shown the action of frequency dependence throughout the fossil record in invasion experiments with SS and FS strains extracted at various time points. In addition, Spencer et al. [28] have already argued in detail why clonal interference is unlikely to be the main driver for the pattern of evolutionary branching observed in population 18, which is one of the populations used for the present study. Clonal interference may have played a role in generating some of the polymorphisms observed within the SS and within the FS lineages, and in the timing of the rise of various mutations. Overall, however, it seems clear that the basic coexistence between the SS and FS ecotypes are not due to clonal interference, but to frequency-dependent ecological interactions. Indeed, similar evidence has led to the conclusion that a polymorphism in one of R. Lenski's long-term experimental lines, Ara-2 [18],[49], evolved as a result of niche construction [15],[50].

It is a hallmark of frequency dependence that one type's abundance creates the niche for another type's invasion. Although we cannot rule out clonal interference, the sequence of alternating invasions observed in the fossil records of our experimental lines is consistent with this process of reciprocal niche construction. In particular, as is apparent from Figures 3 and 4, the rise of the first FS mutations consistently following in the wake of the establishment of first SS mutations is conspicuous, and so is the rise of the SS-associated *nadR* mutations following the appearance of the first FS mutations. We note that the limited replication of this study prevents many rigorous statistical tests, so that many of our results can only be described qualitatively, not quantitatively. With the continuing rapid decline in the cost of sequencing data, it is quickly becoming feasible to carry out studies similar to ours with higher temporal resolution and across larger numbers of populations, which will make rigorous statistical analyses possible.

Nevertheless, it seems unlikely that the consistent pattern of alternating invasions observed in our three lines is due to chance alone, and given that the endpoint FS and SS strains coexist due to frequency dependence, it is tempting to conclude that the patterns of invasion reflect the action of frequency-dependent selection in the course of the evolution experiment. The observed diversification should then be viewed in the light of the theory of adaptive diversification due to frequency-dependent interactions [9]. It is worth noting that much (but not all) of this theory is based on the assumption of many mutations of small effect, and the basic theoretical phenomenon of evolutionary branching in particular is an essentially continuous process in phenotype space [6], which moreover is often presented as a symmetric pattern of diversification. In contrast, in our experimental lines diversification is obviously due to a few mutations of large effect, and the pattern of diversification is asymmetric in phenotype space [28]. However, many aspects of the theory of adaptive diversification are robust to introducing large mutational effects, and asymmetric evolutionary branching is entirely feasible [9]. Therefore, our experimental results can be seen as proof of this robustness, and as providing a full description of adaptive diversification at the genetic level, revealing parallel evolutionary dynamics, and thus a high degree of determinism, in the sympatric origin and subsequent divergence of ecologically distinct lineages.

## Materials and Methods

We isolated clones from frozen samples of populations 18 and 19 from day 156 of the evolution experiment of Spencer et al. [28]. Frozen samples were inoculated into 10 mL of the growth medium, grown overnight at 37°C with shaking, and spread onto agar plates. We arbitrarily chose 10 small colonies and 10 large colonies from each population and measured their growth profiles over 24 h as described in Spencer et al. [28]. From each population, we chose two large colonies with unambiguous SS growth profiles and two small colonies with unambiguous FS growth profiles for sequencing. For population 20, we used previously isolated clones [30], also from day 156 of the experiment. In this experiment, replicate populations were founded from isogenic lines of *E. coli* B and cultured in well-mixed condition for 183 d (∼1,230 generations) with daily (∼6.7 generations) transfers to fresh medium (Text S1). Populations 18 and 20 were initiated with REL606, and population 19 with REL607 [51]. REL606 and REL607 perform similarly in the growth environment of the evolution experiment [28],[51],[52].

For the time point samples, we chose 16 time points corresponding to days 0, 6, 12, 19, 30, 40, 51, 61, 72, 82, 96, 111, 124, 138, 156, and 183 of the evolution experiment for a total of 48 time point samples. We sequenced paired ends of fragments of genomic DNA samples from 12 clones (2 SS and 2 FS from each of three populations) and 48 time point samples (16 time points from each population) on an Illumina HiSeq 2000 using standard procedures. The paired *t* test reported for number of mutations in FS versus SS compared the mean number of mutations in FS clones to that in SS clones from the same population, considering only genealogically independent comparisons (one each for populations 18 and 20, two for population 19, since there were two independent origins of SS in this population).

Paired-end sequencing was performed on an Illumina HiSeq 2000 at the University of British Columbia's Biodiversity Research Centre. The clonal samples were prepared with the Illumina TruSeqTM DNA Sample Preparation Kit, and the time point samples with the NEXTflex DNA Sequencing Kit and DNA Barcodes by Bioo Scientific (Austin, TX). We used CASAVA 1.8 (Illumina, Inc., San Diego, CA) to demultiplex sequencing reads by barcode and generate files in FASTQ format [53] for use in all downstream analyses. All FASTQ files were deposited in the NCBI short read archive (accession: SRP017657). We identified SNPs and small (≤4 bp) indels and estimated their frequencies in the time point samples using both the main public server and local instances of Galaxy (details below) [54]–[56]. To identify larger indels and estimate their frequencies in the time point samples, we used BreSeq version 0.16 [57]. The sequence [58] of the ancestral strain REL606 (GenBank accession number NC_012967.1) was used as the reference for all mutation screens.

FASTQ files were first filtered for quality, retaining only those reads with ≤5 bases with quality scores <20. Reads were aligned to the reference genome using BWA version 0.5.9-r16 [59] with default settings and treating the reads as single-end, and variants were identified using SAMTools version 0.1.12-r862 [60]. For the 60 sequenced samples (12 clones and 48 time points) average coverage (over the 4,629,812 bp of the reference genome) ranged from 72× to 2,500×. For all 60 samples, >99% of the genome was covered by >30 aligned reads.

We report the frequencies of all variants that both appear in more than one time point sample (within the same population) and rise to at least 5% frequency in one or more of the samples. We also report the frequencies of variants that are found in the clonal samples, regardless of their frequency in the time point samples. Variants supported by a single read at a given time point are not reported unless supported by multiple reads in the next time point. We estimated the frequencies of large deletions (>4 bp) by manually inspecting all reads in which ≥10 bp matched each side of the deleted region. In a few cases, we were able to determine linkage between nearby mutations by examining individual Illumina reads that spanned both loci.

To distinguish changes in allele frequency due to selection from those due to drift, we assume an effective population size (*N_e_*) of 3.3×10^7^, as estimated for *E. coli* grown in similar conditions [51]. Under the Wright-Fisher model [61],[62], drift is a Markov chain, which generates a variance in allele frequency of *pq*/*N_e_* after one generation (for haploids). After *t* generations, the variance is *pq*(1 – *e^t^* ^/*Ne*^). If we assume *p = q = *0.5 (which yields the fastest drift), the variance after 82 generations (the average time separating our time point samples) is 6.21×10^−7^ (s.d. = 7.88×10^−4^ or 0.08%). Using the normal approximation of the binomial, the probability that drift causes an allele frequency change ≥1% from one time point sample to the next is less than 1×10^−12^. Thus, even accounting for multiple tests, the possibility that any of the changes in allele frequency that we discuss are caused solely by drift is remote.

## Supporting Information

Figure S1 Frequencies of mutations in the *spoT* gene in each population. (a) population 18. (b) population 19. (c) population 20. Δ, deletion; +, insertion. Numbers following gene names indicate the affected codon within the gene. FS, SS1, etc. in parentheses indicate which clones (if any) have the mutation. The *spoT*-414 mutation is identical in populations 18 and 20. Since it is FS-associated in population 20, it is likely also FS-associated in population 18.
(EPS)

Figure S2 Frequencies of mutations (other than those in *spoT* and *nadR*) not found in clonal samples in each population. (a) population 18. (b) population 19. (c) population 20. Δ, deletion; +, insertion. Numbers following gene names indicate the affected codon within the gene. Note that the highest frequency on the *y*-axis is 0.4.
(EPS)

Text S1 Supplementary information incorporating Supplementary Methods 1 (Materials and Methods details) and Supplementary Results 1 (*nadR* mutations found in the timelines of the fossil record, but not in the sequenced clones).
(DOCX)

Table S1 Mutations detected in all samples and their effect (if known) on the encoded protein. Predicted effects on amino acid sequences are classified as “Frameshift” (e.g., indels of one or two base pairs), substitutions (e.g., “R→H” indicates an arginine residue replaced with a histidine), or synonymous (e.g., “R→R”). Samples are indicated as population number/clone (FS or SS) or time point (TP, indicating that the mutation is found at >5% in one or more time point samples). Numbers after gene names indicate the affected codon. Gene names separated by a forward slash (“/”) indicate a mutation in the intergenic region.
(DOCX)

## Abbreviations

FS: fast switcher
SS: slow switcher

## References

[1] Mayr E (1963) Animal species and evolution. Cambridge, MA: Belknap.

[2] Coyne JA (1992) Genetics and speciation. Nature 355: 511–515. 1741030 10.1038/355511a0

[3] Rice WR, Hostert EE (1993) Laboratory experiments on speciation: what have we learned in 40 years? Evolution 47: 1637–1653. 10.1111/j.1558-5646.1993.tb01257.x 28568007

[4] Turelli M, Barton NH, Coyne JA (2001) Theory and speciation. Trends Ecol Evol 16: 330–343. 11403865 10.1016/s0169-5347(01)02177-2

[5] Schluter D (2001) Ecology and the origin of species. Trends Ecol Evol 16: 372–380. 11403870 10.1016/s0169-5347(01)02198-x

[6] Geritz SAH, Kisdi É, Meszéna G, Metz JAJ (1998) Evolutionarily singular strategies and the adaptive growth and branching of the evolutionary tree. Evol Ecol 12: 35–57.

[7] Dieckmann U, Doebeli M (1999) On the origin of species by sympatric speciation. Nature 400: 354–357. 10432112 10.1038/22521

[8] Doebeli M, Dieckmann U (2000) Evolutionary branching and sympatric speciation caused by different types of ecological interactions. Am Nat 156: S77–S101. 10.1086/303417 29592583

[9] Doebeli M (2011) Adaptive diversification. Princeton, NJ: Monographs in Population Biology, Princeton University Press.

[10] Rainey PB, Travisano M (1998) Adaptive radiation in a heterogeneous environment. Nature 32: 69–72. 10.1038/27900 9665128

[11] Friesen ML, Saxer G, Travisano M, Doebeli M (2004) Experimental evidence for sympatric ecological diversification due to frequency-dependent competition in *Escherichia coli* . Evolution 58: 245–260. 15068343

[12] Tyerman JG, Havard N, Saxer G, Travisano M, Doebeli M (2005) Unparallel diversification in bacterial microcosms. Proc Biol Sci 272: 1393–1398. 16006323 10.1098/rspb.2005.3068 PMC1560327

[13] Kassen R (2002) The experimental evolution of specialists, generalists, and the maintenance of diversity. Science 15: 173–190.

[14] MacLean RC (2005) Adaptive radiation in microbial microcosms. J Evol Biol 18: 1376–1386. 16313450 10.1111/j.1420-9101.2005.00931.x

[15] Rozen DE, Philippe N, Arjan de Visser J, Lenski RE, Schneider D (2009) Death and cannibalism in a seasonal environment facilitate bacterial coexistence. Ecol Lett 12: 34–44. 19019196 10.1111/j.1461-0248.2008.01257.x

[16] Helling RB, Vargas CN, Adams J (1987) Evolution of *Escherichia coli* during growth in a constant environment. Genetics 358: 349–358. 10.1093/genetics/116.3.349 PMC1203146 3301527

[17] Rosenzweig RF, Sharp RR, Treves DS, Adams J (1994) Microbial evolution in a simple unstructured environment: genetic differentiation in *Escherichia coli* . Genetics 137: 903–917. 7982572 10.1093/genetics/137.4.903 PMC1206068

[18] Rozen D, Lenski R (2000) Long-term experimental evolution in *Escherichia coli*. VIII. Dynamics of a balanced polymorphism. Am Nat 155: 24–35. 10657174 10.1086/303299

[19] Maharjan R, Seeto S, Notley-McRobb L, Ferenci T (2006) Clonal adaptive radiation in a constant environment. Science 313: 514–517. 16825532 10.1126/science.1129865

[20] Linn C, Feder JL, Nojima S, Dambroski HR, Berlocher SH, et al (2003) Fruit odor discrimination and sympatric host race formation in *Rhagoletis* . Proc Natl Acad Sci U S A 100: 11490–11493. 14504399 10.1073/pnas.1635049100 PMC208785

[21] Barluenga M, Stölting KN, Salzburger W, Muschick M, Meyer A (2006) Sympatric speciation in Nicaraguan crater lake cichlid fish. Nature 439: 719–723. 16467837 10.1038/nature04325

[22] Savolainen V, Anstett M-C, Lexer C, Hutton I, Clarkson JJ, et al (2006) Sympatric speciation in palms on an oceanic island. Nature 441: 210–213. 16467788 10.1038/nature04566

[23] Ryan PG, Bloomer P, Moloney CL, Grant TJ, Delport W (2007) Ecological speciation in South Atlantic island finches. Science 315: 1420–1423. 17347442 10.1126/science.1138829

[24] Doebeli M, Ispolatov I (2010) Complexity and diversity. Science 328: 494–497. 20413499 10.1126/science.1187468

[25] Metz JAJ, Geritz SAH, Meszéna G, Jacobs FJA, van Heerwaarden JS (1996) Adaptive dynamics, a geometrical study of the consequences of nearly faithful reproduction. In: van Strien SJ, Verduyn Lunel SM, editors. Stochastic and spatial structures of dynamical systems. North Holland, Amsterdam: KNAW Verhandelingen, Vol. 45. pp. 183–231.

[26] Dieckmann U, Law R (1996) The dynamical theory of coevolution: a derivation from stochastic ecological processes. J Math Biol 34: 579–612. 8691086 10.1007/BF02409751

[27] Spencer CC, Bertrand M, Travisano M, Doebeli M (2007) Adaptive diversification in genes that regulate resource use in *Escherichia coli* . PLoS Genet 3: e15 doi:10.1371/journal.pgen.0030015. 17238290 10.1371/journal.pgen.0030015 PMC1779306

[28] Spencer CC, Tyerman JG, Bertrand M, Doebeli M (2008) Adaptation increases the likelihood of diversification in an experimental bacterial lineage. Proc Natl Acad Sci U S A 105: 1585–1589. 18216261 10.1073/pnas.0708504105 PMC2234188

[29] Tyerman JG, Bertrand M, Spencer CC, Doebeli M (2008) Experimental demonstration of ecological character displacement. BMC Evol Biol 8: 34. 18234105 10.1186/1471-2148-8-34 PMC2267161

[30] Le Gac M, Brazas MD, Bertrand M, Tyerman JG, Spencer CC, et al (2008) Metabolic changes associated with adaptive diversification in *Escherichia coli* . Genetics 178: 1049–1060. 18245349 10.1534/genetics.107.082040 PMC2248342

[31] Le Gac M, Doebeli M (2010) Epistasis and frequency dependence influence the fitness of an adaptive mutation in a diversifying lineage. Mol Ecol 19: 2430–2438. 20497320 10.1111/j.1365-294X.2010.04664.x

[32] Monod J (1942) Recherches sur la croissance des cultures bactériennes. Paris: Hermann & cie.

[33] Lenski RE, Travisano M (1994) Dynamics of adaptation and diversification: a 10,000-generation experiment with bacterial populations. Proc Natl Acad Sci U S A 92: 6808–6814. 10.1073/pnas.91.15.6808 PMC44287 8041701

[34] Wichman HA, Badgett MR, Scott LA, Boulianne CM, Bull JJ (1999) Different trajectories of parallel evolution during viral adaptation. Science 285: 422–424. 10411508 10.1126/science.285.5426.422

[35] Stanek MT, Cooper TF, Lenski RE (2009) Identification and dynamics of a beneficial mutation in a long-term evolution experiment with Escherichia coli. BMC Evol Biol 9: 302. 20040094 10.1186/1471-2148-9-302 PMC2806358

[36] Cooper VS, Schneider D, Blot M, Lenski RE (2001) Mechanisms causing rapid and parallel losses of ribose catabolism in evolving populations of *Escherichia coli* B. J Bacteriol 183: 2834–2841. 11292803 10.1128/JB.183.9.2834-2841.2001 PMC99500

[37] Cooper TF, Rozen DE, Lenski RE (2003) Parallel changes in gene expression after 20,000 generations of evolution in *Escherichia coli* . Proc Natl Acad Sci U S A 100: 1072–1077. 12538876 10.1073/pnas.0334340100 PMC298728

[38] Zhang Y-M, Marrakchi H, Rock CO (2002) The FabR (YijC) transcription factor regulates unsaturated fatty acid biosynthesis in *Escherichia coli* . J Biol Chem 277: 15558–15565. 11859088 10.1074/jbc.M201399200

[39] Liu X, De Wulf P (2004) Probing the ArcA-P modulon of *Escherichia coli* by whole genome transcriptional analysis and sequence recognition profiling. J Biol Chem 279: 12588–12597. 14711822 10.1074/jbc.M313454200

[40] Postma PW, Lengeler JW, Jacobson GR (1993) Phosphoenolpyruvate: carbohydrate phosphotransferase systems of bacteria. Microbiol Rev 57: 543–594. 8246840 10.1128/mr.57.3.543-594.1993 PMC372926

[41] Grose JH, Bergthorsson U, Roth JR (2005) Regulation of NAD synthesis by the trifunctional NadR protein of *Salmonella enterica* . J Bacteriol 187: 2774–2782. 15805524 10.1128/JB.187.8.2774-2782.2005 PMC1070365

[42] Woods R, Schneider D, Winkworth CL, Riley MA, Lenski RE (2006) Tests of parallel molecular evolution in a long-term experiment with *Escherichia coli* . Proc Natl Acad Sci U S A 103: 9107–9112. 16751270 10.1073/pnas.0602917103 PMC1482574

[43] Spira B, Hu X, Ferenci T (2008) Strain variation in ppGpp concentration and RpoS levels in laboratory strains of *Escherichia coli* K-12. Microbiology 154: 2887–2895. 18757823 10.1099/mic.0.2008/018457-0

[44] Dettman JR, Rodrigue N, Melnyk AH, Wong A, Bailey SF, et al (2012) Evolutionary insight from whole-genome sequencing of experimentally evolved microbes. Mol Ecol 21: 2058–2077. 22332770 10.1111/j.1365-294X.2012.05484.x

[45] Barrick JE, Lenski RE (2009) Genome-wide mutational diversity in an evolving population of *Escherichia coli* . Cold Springs Harbor Symp Quant Biol 74: 119–129. 10.1101/sqb.2009.74.018 PMC2890043 19776167

[46] Gerrish PJ, Lenski RE (1998) The fate of competing beneficial mutations in an asexual population. Genetica 102–103: 127–144. 9720276

[47] de Visser JA, Rozen DE (2006) Clonal interference and the periodic selection of new beneficial mutations in Escherichia coli. Genetics 172: 2093–2100. 16489229 10.1534/genetics.105.052373 PMC1456385

[48] Kao KC, Sherlock G (2008) Molecular characterization of clonal interference during adaptive evolution in asexual populations of Saccharomyces cerevisiae. Nature Genetics 40: 1499–1504. 19029899 10.1038/ng.280 PMC2596280

[49] Rozen DE, Schneider D, Lenski RE (2005) Long-term experimental evolution in *Escherichia coli*. XIII. Phylogenetic history of a balanced polymorphism. J Mol Evol 61: 171–180. 15999245 10.1007/s00239-004-0322-2

[50] Le Gac M, Plucain J, Hindré T, Lenski RE, Schneider D (2012) Ecological and evolutionary dynamics of coexisting lineages during a long-term experiment with *Escherichia coli* . Proc Natl Acad Sci U S A 109: 9487–9492. 22645336 10.1073/pnas.1207091109 PMC3386082

[51] Lenski RE, Rose MR, Simpson SC, Tadler SC (1991) Long-term experimental evolution in *Escherichia coli*. I. Adaptation and divergence during 2000 generations. Am Nat 138: 1315–1341.

[52] Lenski RE (1988) Experimental studies of pleiotropy and epistasis in *Escherichia coli*. I. Variation in competitive fitness among mutants resistant to virus T4. Evolution 42: 425–432. 10.1111/j.1558-5646.1988.tb04149.x 28564005

[53] Cock PJA, Fields CJ, Goto N, Heuer ML, Rice PM (2010) The Sanger FASTQ file format for sequences with quality scores, and the Solexa/Illumina FASTQ variants. Nucleic Acids Res 38: 1767–1771. 20015970 10.1093/nar/gkp1137 PMC2847217

[54] Goecks J, Nekrutenko A, Taylor J (2010) Galaxy: a comprehensive approach for supporting accessible, reproducible, and transparent computational research in the life sciences. Genome Biol 11: R86. 20738864 10.1186/gb-2010-11-8-r86 PMC2945788

[55] Blankenberg D, Von Kuster G, Coraor N, Ananda G, Lazarus R, et al (2010) Galaxy: a web-based genome analysis tool for experimentalists. Current Protocols in Molecular Biology Chapter 19: Unit 19.10.1–21. 10.1002/0471142727.mb1910s89 PMC4264107 20069535

[56] Giardine B, Riemer C, Hardison RC, Burhans R, Elnitski L, et al (2005) Galaxy: a platform for interactive large-scale genome analysis. Genome Res 15: 1451–1455. 16169926 10.1101/gr.4086505 PMC1240089

[57] Barrick JE, Yu DS, Yoon SH, Jeong H, Oh TK, et al (2009) Genome evolution and adaptation in a long-term experiment with *Escherichia coli* . Nature 461: 1243–1247. 19838166 10.1038/nature08480

[58] Jeong H, Barbe V, Lee CH, Vallenet D, Yu DS, et al (2009) Genome sequences of *Escherichia coli* B strains REL606 and BL21(DE3). J Mol Biol 394: 644–652. 19786035 10.1016/j.jmb.2009.09.052

[59] Li H, Durbin R (2009) Fast and accurate short read alignment with Burrows-Wheeler transform. Bioinformatics 25: 1754–1760. 19451168 10.1093/bioinformatics/btp324 PMC2705234

[60] Li H, Handsaker B, Wysoker A, Fennell T, Ruan J, et al (2009) The Sequence Alignment/Map format and SAMtools. Bioinformatics 25: 2078–2079. 19505943 10.1093/bioinformatics/btp352 PMC2723002

[61] Fisher RA (1930) The genetical theory of natural selection. Oxford, UK: Clarendon Press.

[62] Wright S (1949) Evolution in Mendelian populations. Genetics 16: 97–159. 10.1093/genetics/16.2.97 PMC1201091 17246615

