# Supplementary material for: Parallel Evolutionary Dynamics of Adaptive Diversification in Escherichia coli
Source: PLoS Biol. 2013 Feb 19;11(2):e1001490. doi: 10.1371/journal.pbio.1001490 (PMC3576414; doi:10.1371/journal.pbio.1001490)
Supplement: Text S1 — Supplementary information incorporating Supplementary Methods 1 (Materials and Methods details) and Supplementary Results 1 (nadR mutations found in the timelines of the fossil record, but not in the sequenced clones). (DOCX) [file pbio.1001490.s003.docx]

**Supporting Information**

**1. Methods details**

During the evolution experiment, initially isogenic cultures were incubated at 37°C in 18-mm-diameter test tubes containing 10 ml of liquid DMGA medium (Davis minimal medium supplemented with 250 mg/l glucose and 1323 mg/l sodium acetate trihydrate), shaken continuously at 250 rpm and transferred to fresh medium daily at 1:101 dilution [1]. Samples of each population were frozen in 20% glycerol at -80°C at 3-4 day (~20-27 generation) intervals. To isolate FS and SS clones from populations 18 and 19, we used the frozen samples from day 156 of the experiment to inoculate 10 mL of fresh liquid DMGA medium. Frozen samples were grown in 18 mm test tubes with 10 ml DMGA for 24 hours at 37°C with shaking at 250 rpm, then plated in serial dilution on tryptone agar plates.

For the time point samples, we inoculated 300 mL of DMGA with 100 μl of thawed and mixed bacteria from the frozen samples. For both clonal and time point samples, bacteria were grown for 24 hours at 37°C and shaken continuously at 250 rpm, bacterial cells harvested by centrifugation, and genomic DNA isolated using phenol:chloroform with Phase-lock gels (5Prime, Inc., Gaithersburg, MD) and isopropanol precipitation.

REL607, the founding strain for population 19, differs from REL606 by a single-base substitution in codon 92 of the *araA* gene (bp 70,867) resulting in a change from Gly to Asp in the encoded polypeptide [2]. In addition, we found that a previously undetected single-base substitution in codon 10 of the *recD* gene (bp 2,847,052), resulting in a change from Val to Ala in the encoded polypeptide, was present in all samples sequenced from population 19 (at 100% frequency in all time point samples). Since this substitution was present at 100% frequency in the sample from the first day of the experiment, we assume that it was present in the founding strain.

**2. *nadR* mutations found in the timelines of the fossil record, but not in the sequenced clones**

Various *nadR* mutations were detected in the time point samples, but not in the clones (Fig. 5). Since *nadR* mutations are associated with all of the sequenced SS clones and none of the sequenced FS clones, it seems likely that these uncharacterized *nadR* mutations are also associated with SS ecotypes but not detected because of our limited sampling (i.e., only two SS clones from each population). In particular, it is likely that the *nadR*-290 mutation in population 20 is SS-associated, since the identical mutation in population 19 is SS-associated. Also, the *nadR* Δ13 bp mutation in population 18 (Fig. 5a) must be associated with the SS-ecotype, which can be argued as follows. The frequency data for population 18 shown in Figures 3a and 4a indicate that essentially all bacteria present at the end of the evolution experiment are either SS or FS, and that all the FS bacteria present at that time had the *wecF*-244 mutation. Therefore, if the *nadR* Δ13 bp mutation were not SS-associated, it would have to be in the same genome as the *wecF*-244 mutation. But the *wecF*-244 mutation arose after the *nadR* Δ13 bp mutation in the fossil record (Fig. 2, main text), and hence the *wecF*-244 mutation would have to have arisen on a *nadR* Δ13 bp background, and all bacteria with the *wecF*-244 mutation (including 18-FS1 and 18-FS2) should also have the *nadR* Δ13 bp mutation, which is not the case (Fig. 1).

**References:**

1. Spencer CC, Tyerman JG, Bertrand M, Doebeli M (2008) Adaptation increases the likelihood of diversification in an experimental bacterial lineage. Proc Natl Acad Sci U S A 105: 1585–1589.

2. Studier FW, Daegelen P, Lenski RE, Maslov S, Kim JF (2009) Understanding the differences between genome sequences of *Escherichia coli* B strains REL606 and BL21(DE3) and comparison of the *E. coli* B and K-12 genomes. J Mol Biol 394: 653–680.

**Figure legends:**

**Figure S1.** **Frequencies of mutations in the *spoT* gene in each population.** **a,** population 18. **b,** population 19. **c,** population 20. “Δ” indicates a deletion; “+” indicates an insertion. Numbers following gene names indicate the affected codon within the gene. FS, SS1, etc. in parentheses indicate which clones (if any) have the mutation. The *spoT*-414 mutation is identical in populations 18 and 20. Since it is FS-associated in population 20, it is likely also FS-associated in population 18.

**Figure S2.** **Frequencies of mutations (other than those in *spoT* and *nadR*) not found in clonal samples in each population.** **a,** population 18. **b,** population 19. **c,** population 20. “Δ” indicates a deletion; “+” indicates an insertion. Numbers following gene names indicate the affected codon within the gene. Note that the highest frequency on the y-axis is 0.4.
