# Supplementary material for: Parallel Evolutionary Dynamics of Adaptive Diversification in Escherichia coli
Source: PLoS Biol. 2013 Feb 19;11(2):e1001490. doi: 10.1371/journal.pbio.1001490 (PMC3576414; doi:10.1371/journal.pbio.1001490)
Supplement: Table S1 — Mutations detected in all samples and their effect (if known) on the encoded protein. Predicted effects on amino acid sequences are classified as “Frameshift” (e.g., indels of one or two base pairs), substitutions (e.g., “R→H” indicates an arginine residue replaced with a histidine), or synonymous (e.g., “R→R”). Samples are indicated as population number/clone (FS or SS) or time point (TP, indicating that the mutation is found at >5% in one or more time point samples). Numbers after gene names indicate the affected codon. Gene names separated by a forward slash (“/”) indicate a mutation in the intergenic region. (DOCX) [file pbio.1001490.s004.docx]

**Table S1.** Mutations detected in all samples and their effect (if known) on the encoded protein. Predicted effects on amino acid sequences are classified as “Frameshift” (e.g. indels of one or two base pairs), substitutions (e.g. “R🡪H indicates an arginine residue replaced with a histidine), or synonymous (e.g. “R🡪R”). Samples are indicated as population number / clone (FS or SS) or time point (TP, indicating that the mutation is found at >5% in one or more time point samples). Numbers after gene names indicate the affected codon. Gene names separated by a forward slash (“/”) indicate a mutation in the intergenic region.

|  |  |  |  |  | Sample | | | | | | | | | | | | | | |
| --- | --- | --- | --- | --- | --- | --- | --- | --- | --- | --- | --- | --- | --- | --- | --- | --- | --- | --- | --- |
| Starting |  |  |  |  | 18 | 18 | 18 | 18 | 18 | 19 | 19 | 19 | 19 | 19 | 20 | 20 | 20 | 20 | 20 |
| position |  | Mutation | Effect | Gene | SS1 | SS2 | FS1 | FS2 | TP | SS1 | SS2 | FS1 | FS2 | TP | SS1 | SS2 | FS1 | FS2 | TP |
| 198,469 |  | CGT-->CAT | R-->H | *uppS*-242 |  |  |  |  |  |  |  | X | X | X |  |  |  |  |  |
| 387,800 |  | T-->G |  | *phoR*/*brnQ* |  |  |  |  |  |  |  |  |  |  |  |  |  | X |  |
| 732,435 |  | Δ 3bp |  | *ybgQ* |  |  |  |  |  |  |  |  |  |  |  |  |  |  | X |
| 743,404 |  | ATC-->ATA | I-->I | *sucB*-364 |  |  |  |  | X |  |  |  |  |  |  |  |  |  |  |
| 1,027,036 |  | C-->A | A-->E | *rlmL*-701 |  |  |  |  |  |  |  |  |  |  |  |  | X |  |  |
| 1,131,745 |  | Δ 2bp |  | *yceA* |  |  |  |  |  |  |  |  |  |  |  |  | X | X | X |
| 1,172,374 |  | +6bp |  | *ycfH*/*ptsG* |  |  |  |  |  |  |  | X | X | X |  |  |  |  |  |
| 1,173,450 |  | TCG-->TGG | S-->W | *ptsG*-338 |  |  |  |  |  |  |  |  |  |  |  |  | X | X | X |
| 1,230,056 |  | Δ T |  | *ycgN*/*umuD* |  |  |  |  |  |  |  |  |  |  |  |  | X | X | X |
| 1,235,793 |  | Δ A | Frameshift | *fadR* |  |  |  |  |  |  |  |  |  | X |  |  |  |  |  |
| 1,240,901 |  | Δ 4bp | Frameshift | *ycgO* |  |  |  |  |  |  |  | X | X | X |  |  |  |  |  |
| 1,330,866 |  | GTT-->TTT | V-->F | *topA*-483 |  |  |  |  |  |  |  |  |  | X |  |  |  |  |  |
| 1,462,251 |  | IS150 |  | *mokB*/*trg* |  |  |  |  |  |  |  |  |  |  |  | X |  |  |  |
| 1,504,662 |  | GAC-->GGC | D-->G | *ydcD*-60 |  |  |  |  |  |  |  |  |  |  | X |  |  |  |  |
| 1,816,118 |  | ATT-->ATA | I-->I | *ynjD*-48 |  |  |  |  |  |  |  |  |  |  | X |  |  |  |  |
| 2,356,984 |  | +T |  | *yfbV*/*ackA* |  |  | X | X | X |  |  | X | X | X |  |  |  |  |  |
| 2,359,510 |  | GCG-->GTG | A-->V | *pta*-391 |  |  |  |  |  |  |  |  |  |  |  |  | X | X | X |
| 2,359,920 |  | ATC-->TTC | I-->F | *pta*-528 |  |  |  |  |  |  |  |  |  | X |  |  |  |  |  |
| 2,448,493 |  | IS186 |  | *nupC*/*yfeA* |  |  |  |  |  |  |  |  |  |  |  |  |  | X |  |
| 2,612,293 |  | G-->A |  | *yfhK*/*purL* |  |  |  | X |  |  |  |  |  |  |  |  |  |  |  |
| 2,643,895 |  | GAT-->GTT | D-->V | *yfiQ*-817 |  |  |  |  | X |  |  |  |  |  |  |  |  |  |  |
| 2,861,563 |  | CGT-->TGC | R-->C | *ptsP*-498 |  |  |  |  |  |  |  |  |  | X |  |  |  |  |  |
| 2,872,155 |  | Δ C |  | *galR* |  |  |  |  |  |  |  |  |  |  |  |  |  |  | X |
| 3,055,070 |  | T-->G |  | *glcD*/*glcC* |  |  |  |  |  |  |  |  |  |  |  |  |  |  | X |
| 3,105,536 |  | CTC-->CGC | L-->R | *ygiY*-294 |  |  |  |  |  |  |  |  |  | X |  |  |  |  |  |
| 3,241,814 |  | GCA-->GCC | A-->A | *deaD*-427 |  |  |  |  |  |  |  | X | X | X |  |  |  |  |  |
| 3,312,640 |  | Δ 9bp |  | *argR* |  |  |  |  |  |  |  | X | X | X |  |  |  |  |  |
| 3,328,633 |  | GTT-->CTT | V-->L | *mreB*-116 |  |  |  |  |  |  |  |  |  |  |  |  |  |  | X |
| 3,338,110 |  | +T |  | *prmA*/*yhdG* |  |  |  |  |  |  |  |  |  |  |  |  |  |  | X |
| 3,339,151 |  | +C |  | *yhdG*/*fis* |  |  |  |  |  |  |  |  |  |  |  |  |  |  | X |
| 3,339,406 |  | GGC-->GAC | G-->D | *fis*-82 |  |  |  |  |  |  |  |  |  |  |  |  |  |  | X |
| 3,403,946 |  | CTT-->ATT | L-->I | *yheO*-134 |  |  |  |  |  |  |  |  |  |  |  |  |  |  | X |
| 3,761,703 |  | GTG-->GAG | V-->E | *spoT*-316 |  |  |  |  |  |  |  |  |  |  |  |  |  |  | X |
| 3,761,745 |  | ATG-->ACG | M-->T | *spoT*-330 |  |  |  |  |  |  |  |  |  | X |  |  |  |  |  |
| 3,761,862 |  | GCC-->GTC | A-->V | *spoT*-369 |  |  | X | X | X |  |  |  |  |  |  |  |  |  |  |
| 3,761,934 |  | CCG-->CAG | P-->Q | *spoT*-393 |  |  |  |  | X |  |  |  |  |  |  |  |  |  |  |
| 3,761,996 |  | CAT-->AAT | H-->N | *spoT*-414 |  |  |  |  | X |  |  |  |  |  |  |  | X | X | X |
| 3,762,080 |  | ACC-->CCC | T-->P | *spoT*-442 |  |  |  |  |  | X |  |  |  | X |  |  |  |  |  |
| 3,762,116 |  | AAT-->CAT | N-->H | *spoT*-454 |  |  |  |  |  |  |  |  |  |  | X | X |  |  | X |
| 3,762,122 |  | GCT-->CCT | A-->P | *spoT*-456 |  |  |  |  |  |  |  |  |  | X |  |  |  |  |  |
| 3,762,167 |  | CGT-->AGT | R-->S | *spoT*-471 | X | X |  |  | X |  |  |  |  |  |  |  |  |  |  |
| 3,762,525 |  | AAA-->ACA | K-->T | *spoT*-590 |  |  |  |  |  |  | X |  |  | X |  |  |  |  |  |
| 3,762,857 |  | CGA-->TGA | R-->* | *spoT*-701 |  |  |  |  |  |  |  |  |  | X |  |  |  |  |  |
| 3,814,706 |  | Δ 4bp | Frameshift | *emrD* |  |  |  |  |  |  |  | X | X | X |  |  |  |  |  |
| 3,825,074 |  | GGT-->AGT | G-->S | *yidE*-521 |  |  |  |  |  |  |  |  |  | X |  |  |  |  |  |
| 3,894,997 |  | Δ155 bp |  | *rbs* operon |  |  |  |  |  | X |  |  |  | X |  |  |  |  |  |
| 3,894,997 |  | Δ1160 bp |  | *rbs* operon |  |  |  |  |  |  |  |  |  |  | X | X |  |  | X |
| 3,894,997 |  | Δ4600 bp |  | *rbs* operon |  |  |  |  |  |  | X |  |  | X |  |  |  |  |  |
| 3,894,997 |  | Δ5797 bp |  | *rbs* operon | X | X |  |  | X |  |  |  |  |  |  |  |  |  |  |
| 3,894,997 |  | Δ6869 bp |  | *rbs* operon |  |  |  |  |  |  |  |  |  |  |  |  |  | X |  |
| 3,894,997 |  | Δ8243 bp |  | *rbs* operon |  |  |  |  |  |  |  | X | X | X |  |  |  |  |  |
| 3,928,261 |  | ATC-->ATG | I-->M | *rho*-15 |  |  |  |  |  |  |  |  |  |  |  |  | X | X | X |
| 3,941,131 |  | GCG-->GTG | A-->V | *wecF*-244 |  |  | X | X | X |  |  |  |  |  |  |  |  |  |  |
| 3,941,305 |  | CCT-->CTT | P-->L | *wecF*-302 |  |  |  |  | X |  |  |  |  |  |  |  |  |  |  |
| 4,141,016 |  | ACC-->AAC | T-->N | *yijc*-30 |  |  | X | X | X |  |  |  |  |  |  |  |  |  |  |
| 4,201,750 |  | TAC-->TAA | Y-->* | *iclR*-270 |  |  |  |  |  |  |  |  |  |  |  |  |  |  | X |
| 4,202,400 |  | GGG-->GTG | G-->R | *iclR*-54 |  |  |  |  |  |  |  |  |  |  |  |  |  |  | X |
| 4,202,456 |  | Δ 10 bp | Frameshift | *iclR* |  |  | X | X | X |  |  |  |  |  |  |  |  |  |  |
| 4,436,314 |  | CCG-->CTG | P-->L | *ytfT*-164 |  |  |  |  |  | X |  |  |  |  |  |  |  |  |  |
| 4,438,213 |  | GGG-->GTG | G-->V | *fbp*-216 |  |  |  |  |  |  |  |  |  |  |  |  |  |  | X |
| 4,503,322 |  | A-->G |  | *fecI*/*insA-25* |  |  | X | X | X |  |  |  |  |  |  |  |  |  |  |
| 4,505,393 |  | Δ 34k bp |  | *mcrC*-*yijN* |  |  |  |  |  |  |  |  | X | X |  |  |  |  |  |
| 4,613,772 |  | GCC-->ACC | A-->T | *serB*-222 |  |  |  |  |  |  | X |  |  |  |  |  |  |  |  |
| 4,615,623 |  | TTA-->TGA | L-->* | *nadR*-32 |  |  |  |  | X |  |  |  |  |  |  |  |  |  |  |
| 4,615,687 |  | +T | Frameshift | *nadR*-53 |  |  |  |  |  |  |  |  |  | X |  |  |  |  |  |
| 4,615,687 |  | Δ T | Frameshift | *nadR*-53 |  |  |  |  |  |  |  |  |  | X |  |  |  |  |  |
| 4,615,693 |  | ΔG | Frameshift | *nadR*-55 |  |  |  |  |  |  | X |  |  | X |  |  |  |  |  |
| 4,615,772 |  | TAC-->CAC | Y-->H | *nadR*-82 |  |  |  |  |  |  |  |  |  |  |  |  |  |  | X |
| 4,615,818 |  | ATT-->AGT | I-->S | *nadR*-97 | X | X |  |  |  |  |  |  |  |  |  |  |  |  |  |
| 4,616,036 |  | IS186 |  | *nadR* IS186 |  |  |  |  |  |  |  |  |  |  | X |  |  |  |  |
| 4,616,160 |  | ATC-->AAC | I-->N | *nadR*-211 |  |  |  |  | X |  |  |  |  |  |  |  |  |  |  |
| 4,616,235 |  | Δ13 bp |  | *nadR* |  |  |  |  | X |  |  |  |  |  |  |  |  |  |  |
| 4,616,396 |  | GGC-->AGC | G-->S | *nadR*-290 |  |  |  |  |  | X |  |  |  | X |  |  |  |  | X |
| 4,616,397 |  | GGC-->GAC | G-->D | *nadR*-290 |  |  |  |  | X |  |  |  |  |  |  |  |  |  |  |
| 4,616,399 |  | CAC-->TAC | H-->Y | *nadR*-291 |  |  |  |  |  |  |  |  |  |  |  |  |  |  | X |
| 4,616,409 |  | TAC-->TGC | Y-->C | *nadR*-294 |  |  |  |  | X |  |  |  |  |  |  |  |  |  | X |
| 4,616,410 |  | TAC-->TAA | Y-->* | *nadR*-294 |  |  |  |  |  |  |  |  |  | X |  |  |  |  |  |
| 4,616,476 |  | ΔG |  | *nadR*-316 |  |  |  |  |  |  |  |  |  | X |  |  |  |  |  |
| 4,616,708 |  | ΔC |  | *nadR*-394 |  |  |  |  |  |  |  |  |  |  |  | X |  |  | X |
| 4,628,226 |  | ACT-->GCT | T-->A | *arcA*-81 |  |  |  |  |  |  |  |  |  |  |  |  | X | X | X |
| 4,628,240 |  | GCG-->GAG | A-->E | *arcA*-76 |  |  |  |  |  |  |  |  |  |  |  |  |  |  | X |
